# A Knowledge-Enhanced Platform (MetaSepsisKnowHub) for Retrieval Augmented Generation–Based Sepsis Heterogeneity and Personalized Management: Development Study

**DOI:** 10.2196/67201

**Published:** 2025-06-06

**Authors:** Chi Zhang, Hao Yang, Xingyun Liu, Rongrong Wu, Hui Zong, Erman Wu, Yi Zhou, Jiakun Li, Bairong Shen

**Affiliations:** 1 Joint Laboratory of Artificial Intelligence for Critical Care Medicine Department of Critical Care Medicine and Institutes for Systems Genetics, Frontiers Science Center for Disease-related Molecular Network West China Hospital, Sichuan University Chengdu China; 2 Information Center and Department of Critical Care Medicine and Institutes for Systems Genetics West China Hospital, Sichuan University Chengdu China; 3 Department of Computer Science and Information Technologies University of A Coruña, Research Center for Information and Communications Technologies, Biomedical Research Institute of A Coruña, Iberian Society of Telehealth and Telemedicine A Coruña Spain; 4 Department of Urology West China Hospital, Sichuan University Chengdu China

**Keywords:** human sepsis, knowledge-enhanced, personalized application, retrieval augmented generation, precision medicine

## Abstract

**Background:**

Sepsis is a severe syndrome of organ dysfunction caused by infection; it has high heterogeneity and high in-hospital mortality, representing a grim clinical challenge for precision medicine in critical care.

**Objective:**

We aimed to extract reported sepsis biomarkers to provide users with comprehensive biomedical information and integrate retrieval augmented generation (RAG) and prompt engineering to enhance the accuracy, stability, and interpretability of clinical decisions recommended by large language models (LLMs).

**Methods:**

To address the challenge, we established and updated the first knowledge-enhanced platform, MetaSepsisKnowHub, comprising 427 sepsis biomarkers and 423 studies, aiming to systematically collect and annotate sepsis biomarkers to guide personalized clinical decision-making in the diagnosis and treatment of human sepsis. We curated a tailored LLM framework incorporating RAG and prompt engineering and incorporated 2 performance evaluation scales: the System Usability Scale and the Net Promoter Score.

**Results:**

The overall quantitative ratings of expert-reviewed clinical recommendations based on RAG surpassed baseline responses generated by 4 LLMs and showed a statistically significant improvement in textual questions (GPT-4: mean 75.79, SD 7.11 vs mean 81.59, SD 9.87; *P*=.02; GPT-4o: mean 70.36, SD 7.63 vs mean 77.98, SD 13.26; *P*=.02; Qwen2.5-instruct: mean 77.08 SD 3.75 vs mean 85.46, SD 7.27; *P*<.001; and DeepSeek-R1: mean 77.67, SD 3.66 vs mean 86.42, SD 8.56; *P*<.001), but no significant statistical differences could be measured in clinical scenarios. The RAG assessment score comparing RAG-based responses and expert-provided benchmark answers illustrated prominent factual correctness, accuracy, and knowledge recall compared to the baseline responses. After use, the average the System Usability Scale score was 82.20 (SD 14.17) and the Net Promoter Score was 72, demonstrating high user satisfaction and loyalty.

**Conclusions:**

We highlight the pioneering MetaSepsisKnowHub platform, and we show that combining MetaSepsisKnowHub with RAG can minimize limitations on precision and maximize the breadth of LLMs to shorten the bench-to-bedside distance, serving as a knowledge-enhanced paradigm for future application of artificial intelligence in critical care medicine.

## Introduction

### Background

Sepsis, defined as life-threatening organ dysfunction caused by a dysregulated host response to infection, is one of the leading causes of human mortality and disability worldwide and accounts for an in-hospital death rate of 10% to 20% [1-3]. Sepsis, along with septic shock, is an important clinical challenge faced by critical care medicine. Every year, the number of patients with sepsis worldwide exceeds 48.9 million, of whom 11 million die, with a mortality rate of more than 1 in 5 [4,5]. However, there is a lack of personalized and precise diagnosis and treatment for sepsis, as sepsis is a complex, heterogeneous, and nonspecific syndrome [6].

Biomarkers, indicators of biological states or conditions, have been widely used to improve the diagnosis, treatment, and prognosis of human sepsis [7,8]. Due to the heterogeneity of sepsis, the reported biomarkers for human sepsis are very diverse and inconsistent with each other [9,10]. The knowledge distributed in publications is huge but isolated and unstructured. The extraction of useful information from such a large number of studies is extraordinarily time-consuming. Moreover, the information contained in different papers is not standardized [11,12]. Intensivists find it challenging to implement personalized clinical applications of biomarkers to aid accurate and explainable diagnosis and treatment of sepsis. Thus, based on a knowledge-guided, data-driven scientific discovery paradigm for complex systems, there is an urgent need for an integrated knowledge platform to collect, classify, annotate, analyze, and display biomedical information on sepsis biomarkers; to shape diagnostic, therapeutic, and prognostic laboratory bench practices; and shorten the distance between bench and bedside while considering the heterogeneity of human sepsis [13,14]. Generative artificial intelligence (GenAI) has introduced disruptive innovations in internal medicine and surgery, yet it faces significant challenges, including bias, lack of transparency, inaccurate information, and static knowledge, which limit its broader application [15,16]. Retrieval augmented generation (RAG) is a prompt engineering architecture integrating external knowledge retrieval with GenAI to produce accurate, transparent, and evidence-based responses. In critical care medicine, RAG demonstrates significant potential to improve diagnostic reliability and therapeutic personalization through the real-time integration of domain-specific clinical knowledge. Furthermore, leveraging real-time, evidence-based data enables RAG to support the clinical application of GenAI, advancing personalized sepsis care and facilitating more informed, accurate, and dynamic decision-making in sepsis management [17-19].

### This Study

Herein, we established the first sepsis biomarker database [20] and updated it to version 2.0 for constructing a comprehensive knowledge-enhanced platform, MetaSepsisKnowHub [21], to assist intensivists in personalized medicine for human sepsis. MetaSepsisKnowHub contains annotated biomedical information on 427 sepsis biomarkers collected from PubMed and Web of Science databases, which were collected by manual text mining. The selection criteria were approved by relevant experts. All biomarkers were annotated in internationally recognized knowledge databases (including NCBI Gene, Protein database, Ensembl Genome Browser, UniProt, and Online Mendelian Inheritance in Man) and classified into different groups according to disease stratification, application type, biomarker category, effectiveness, validation, level of biological effect, sample type, and technique used in each study. Compared to the first version, new additions included attached annotated biomedical information on potential therapeutic targets and corresponding drugs; we also introduced RAG and prompt engineering to enhance the accuracy, similarity, and relevancy of generative clinical decision recommendations of large language models (LLMs) for personalized clinical application and deep phenotyping of human sepsis.

## Methods

### Platform Design

The establishment of MetaSepsisKnowHub, a sepsis biomarker knowledge-enhanced platform, adhered strictly to the biomedical informatics research paradigm. In the early stages of platform design, we conducted comprehensive user demand analysis through semistructured focus group interviews. Participants in the focus group interviews were selected based on their expertise and experience in sepsis diagnosis, prognosis, and management, and included molecular biologists, medical researchers, clinicians, epidemiologists, biostatisticians, and bioinformatics experts [22]. We sought a diverse group of professionals at various stages of research and clinical practice to ensure the platform addressed the needs of all potential users. Participants were invited from prestigious academic institutions, hospitals, and research organizations, with a particular focus on those actively involved in sepsis-related research or clinical decision-making.

During the semistructured interviews, we posed targeted questions to obtained in-depth insights and specific requirements for the platform, including the types of biomarkers most relevant to sepsis management and the preferred formats for data presentation [23]. The semistructured discussions in the focus groups were meticulously recorded and analyzed, with key findings and suggestions incorporated into the platform’s design. As a result, valuable user feedback informed the design scheme of the platform, ensuring that MetaSepsisKnowHub met the practical needs of its intended users and contributed to advancing personalized sepsis care.

### Literature Search

All the included biomedical information in MetaSepsisKnowHub was collected from PubMed and Web of Science databases by manual text mining. The following keywords were used for searching: “(sepsis [Title/Abstract] OR septic shock [Title/Abstract]) AND (biomarker [Title/Abstract] OR marker [Title/Abstract] OR indicator [Title/Abstract]) AND 1900/01/01:2023/07/31 [PDAT].” A total of 6997 original articles were identified based on these criteria.

### Inclusion and Exclusion Criteria

All publications were searched until July 31, 2023. We excluded reviews, case reports, letters, editorials, comments, books, and documents and included 1361 original studies. Articles without full texts, non-English publications, and relevant literature that did not involve human sepsis or biomarkers in the original studies were also excluded. Through intensive text mining of the selected literature, 423 (31.08%) complete English original studies associated with biomarkers of human sepsis met the inclusion and exclusion criteria in total. We finally screened the original data related to biomarkers in the aforementioned articles and extracted 427 different sepsis biomarkers with a total of 644 records (Figure S1 in Multimedia Appendix 1). All sepsis biomarkers included in MetaSepsisKnowHub are derived from peer-reviewed clinical or basic research. The biomarkers we have collected are broad in scope, including genes, RNA, proteins, clinical assessment tools, and composite biomarkers. The categories of biomarkers are annotated in the “Biomarker Category” entry within the knowledge platform. When a study reported multiple sepsis-related biomarkers or when the same sepsis biomarker may encompass different disease severities, application types, and other situations that cannot be consolidated, each biomarker was collected separately and listed individually on the platform.

### Platform Framework

After screening the literature, biomedical information from the research was defined, collected, and organized, including its features, described information, annotation information, application effectiveness, and information about potential therapeutic targets (Table S1 in Multimedia Appendix 1). We categorized the aforementioned biomedical information in a detailed and appropriate manner according to terminology definitions and application orientation and presented the platform framework comprehensively in an entity relationship diagram (Figure S2 in Multimedia Appendix 1).

### Platform Establishment

After data retrieval and processing, we used a browser-server structure and a WAMP system (Windows Server 2016 + Apache 2.4.39 + MySQL [10.4.6-MariaDB] + PHP 7.3.8) to visualize the MetaSepsisKnowHub (Figure S3 in Multimedia Appendix 1). Users can use their own browsers to access MetaSepsisKnowHub conveniently, without installing other components. HTML and CSS were used to create web pages and display interface information. PHP and JavaScript can be used to connect to the knowledge platform and realize the search function. The data are stored in a MySQL database, making it convenient for users to access the platform quickly. We provided the end point, parameters, request examples, and error handling for the application programming interface (API) of MetaSepsisKnowHub on the website for users to access the knowledge. All matching and analyzing operations were implemented on the Windows operating system.

### Annotation

All the included information were annotated by the NCBI Gene, Protein database, Ensembl Genome Browser, UniProt, and Online Mendelian Inheritance in Man database [24-26]. Moreover, MetaSepsisKnowHub systematically annotated sepsis biomarkers in Therapeutic Target Database (TTD) [27,28] and PubChem database [29,30], aiming to identify biomarkers that could serve as potential therapeutic targets for sepsis management.

### Clinical Heterogeneity Analyses

MicroRNA (miRNA) biomarkers can be annotated by miRWalk and users can construct a miRNA-gene interaction network by miRBase after annotation [31-34]. The protein-protein interaction (PPI) analysis was used from the STRING database [35,36]. Enrichment analyses were conducted within Gene Ontology (GO) and Kyoto Encyclopedia of Genes and Genomes (KEGG) [37-40] for sepsis biomarkers.

### Platform Validation and Performance Evaluation

#### RAG-Based LLM Framework Construction

RAG aims to address the challenges faced by knowledge retrieval systems based on LLMs, particularly in fields such as health care. These challenges include timeliness, accuracy, and coverage of knowledge, as well as the need for interactive learning and personalized responses [41]. Our research organized precise, in-depth medical knowledge into question-answer pairs and used embedding API to convert textual and tabular knowledge into vectors, encoding semantic information from the text or tables to match with question vectors. By leveraging the LangChain framework, we created complex prompts, managed prompt engineering, handled human-machine dialogue processes, and integrated diverse data sources. When users pose queries, it is also converted into vectors via the embedding API and compared with the vectors of the knowledge segments using methods such as cosine similarity to find the most relevant text passage. The LangChain framework then helps users combine the most similar passages with the question to form a new prompt, which is sent to embedding API for the GPT-4, GPT-4o, Qwen2.5-instruct, and DeepSeek-R1 models to generate a response. The generated response is further optimized and filtered by LangChain to ensure quality and relevance. This approach, combining LangChain and embedding API, constructs an efficient, accurate, flexible, and scalable RAG-based LLM knowledge retrieval framework. It improves retrieval accuracy while maintaining consistent, high-quality responses and better understanding and answering user-provided queries.

#### Platform Validation

We designed 50 questions, including 25 text-based questions and 25 clinical scenario questions, and posed them as queries to 4 LLMs (including GPT-4, GPT-4o, Qwen2.5-instruct, and DeepSeek-R1) and RAG-based LLM frameworks. We also designed a prompt pattern that instructed the LLMs to generate structured responses (Multimedia Appendix 1). The text-based questions mainly focused on related performance indicators (such as sensitivity, specificity, and cutoff values) of sepsis biomarkers and their ability to assist in the diagnosis of sepsis, evaluating the depth and accuracy of answers generated by the LLMs. On the other hand, the clinical scenario questions mainly concentrated on the early diagnosis and treatment recommendations for patients with suspected sepsis, evaluating the generalization ability of the LLMs and breadth of the generated answers.

To evaluate the accuracy of the answers, we invited 2 clinical experts in critical care medicine to independently score the answers manually, and the final score was the average of the 2 scores. In addition, we used RAG assessments (RAGAs) in conjunction with LlamaIndex for automated assessment. RAGAs is a framework for reference-free evaluation of RAG pipelines [42]. The evaluation focuses on several aspects: faithfulness, answer relevance, context relevance, context recall, context precision, and answer correctness based on factual accuracy and semantic similarity. Finally, we obtained radar charts of evaluation results in RAG application performance.

LLMs generate seemingly plausible content that is actually incorrect, irrelevant to the input prompt, or even contradictory. These phenomena are termed “hallucinations.” There are 3 types of hallucinations in LLMs: input-conflict hallucination (ICH), context-conflict hallucination (CCH), and fact-conflict hallucinations (FCH). ICHs occur when the generated content does not align with the user-provided input. CCHs refer to content generated that contradicts previously generated information. FCHs involve content generated that contradicts established world knowledge [43]. In this study, 2 critical care clinical experts were invited to meticulously and manually examine the completeness, applicability, and types of hallucinations present in 400 responses generated by LLMs.

#### Performance Evaluation

We introduced the System Usability Scale (SUS) [44] and the Net Promoter Score (NPS) [45] to assess usability and user loyalty of MetaSepsisKnowHub. The questionnaires were administered to 25 prospective users, more than 75% (n=19) of whom were clinicians and the vast majority (14/19, 74%) of whom were critical care clinicians. We calculated the SUS and NPS based on feedback and scoring algorithms.

### Statistical Analysis

We used an independent-sample 2-tailed *t* test to compare accuracy between baseline and experimental answers qualitatively and performed Pearson correlation analyses to test the consistency of evaluations from 2 experts. We considered a 2-tailed *P*<.05 to be statistically significant. To conduct statistical analysis, we used Python (version 3.11; Python Software Foundation) and SciPy (version 1.11).

### Ethical Considerations

This study was approved by the Medical Ethics Committee of West China Hospital, Sichuan University (2024-126) in accordance with the Helsinki Declaration. Informed consent was waived by our institutional review board because of the retrospective nature of our study. All data used in the study are anonymized to ensure participant privacy and confidentiality. Protective measures, including deidentification and secure data handling, were implemented to safeguard patient information. No compensation was provided to participants as this study did not involve direct interaction with subjects.

## Results

### Samples

We included original studies to extract accurate biomedical information and raw data on sepsis biomarkers, excluding publication type and language that did not meet the inclusion criteria. Further screening for eligibility of full texts excluded articles with irrelevant content or inaccessible data. Eventually, 427 sepsis biomarkers and 423 original studies were included in MetaSepsisKnowHub.

### Website Interface of MetaSepsisKnowHub

MetaSepsisKnowHub features an intuitive and well-structured website interface designed to facilitate seamless access to the knowledge of sepsis biomarkers. The platform is organized into 6 key sections, as described in Textbox 1.

**Key sections of the MetaSepsisKnowHub platform.**

- Home page: provides a comprehensive introduction to the MetaSepsisKnowHub platform, outlining its purpose, key functionalities, and significance in advancing sepsis research.
- Biomarkers page: showcases a curated repository of sepsis biomarkers, offering detailed biomedical insights, including their diagnostic and prognostic relevance.
- Download page: enables users to access and download biomarker datasets for further analysis, supporting data-driven research and clinical exploration.
- Documents page: serves as a knowledge hub, offering in-depth guidelines on sepsis pathophysiology, platform use, and analytic methodologies. This section also stores statistical summaries and systems biological analyses.
- Submission page: provides a dedicated portal for researchers to contribute new biomarker discoveries, fostering collaborative knowledge expansion.
- Application programming interface (API) documentation: provides users with detailed API end points, parameter examples, code, and data return format references.
- About Us page: introduces the research team behind MetaSepsisKnowHub, highlighting their expertise, contributions, and commitment to advancing sepsis diagnostics and prognostics.

**Figure 1:**
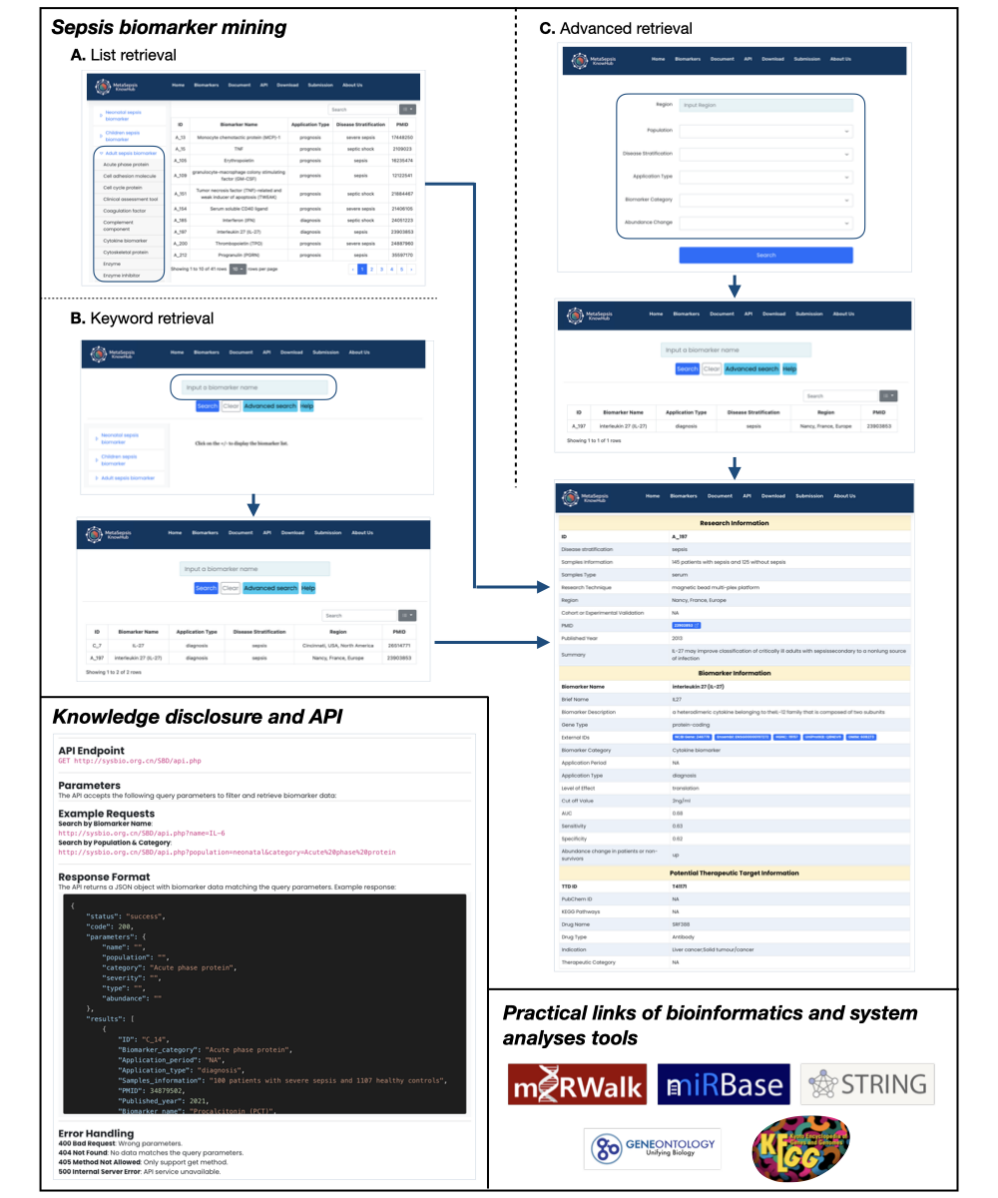
Schematic diagram of the front-end user interface of MetaSepsisKnowHub. (A) List retrieval; (B) keyword retrieval; and (C) advanced retrieval. API: application programming interface.

Each section is meticulously designed to ensure clarity, usability, and scientific rigor, making MetaSepsisKnowHub an indispensable resource for researchers and clinicians seeking to enhance their understanding of sepsis biomarkers (Figure 1).

MetaSepsisKnowHub offers 3 retrieval methods, and users can search and mine sepsis biomarkers by lists and keywords or apply advanced filters like region, disease severity, and biomarker category. Figure 1 outlines how to query sepsis biomarkers via API, with example requests and responses. MetaSepsisKnowHub also provides links to bioinformatics and systems biology tools including miRWalk, miRBase, STRING, GO, and KEGG for further analyses and research supports.

### Application Scenarios of MetaSepsisKnowHub

#### Overview

**Figure 2:**
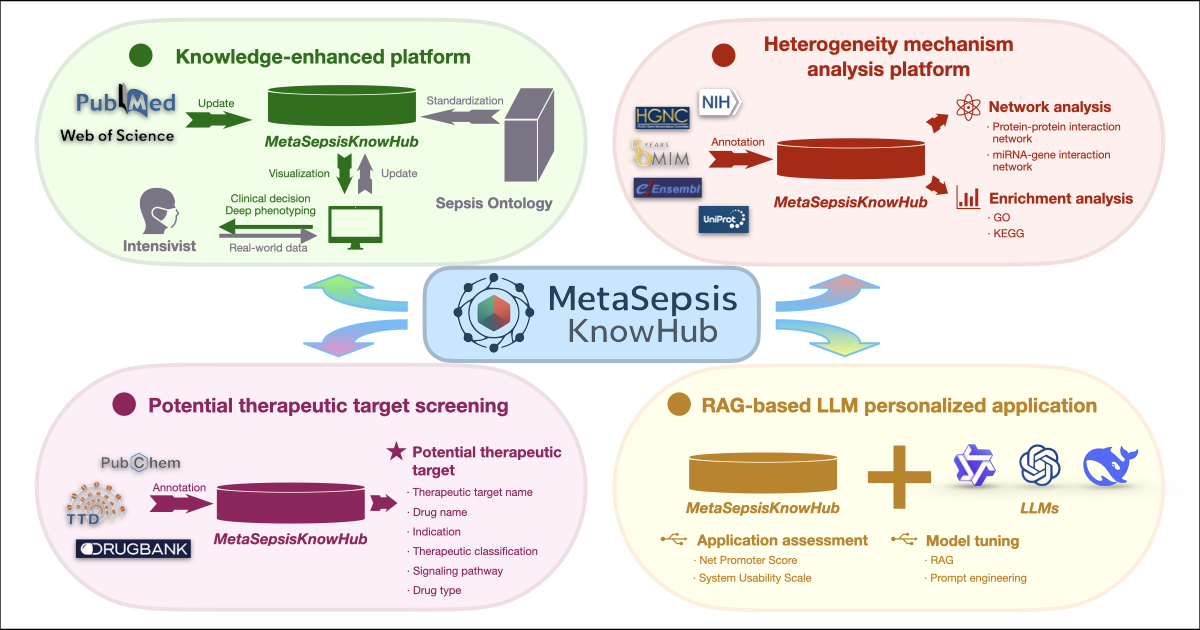
Overview of diverse application scenarios for MetaSepsisKnowHub. GO: Gene Ontology; KEGG: Kyoto Encyclopedia of Genes and Genomes; LLM: large language model; RAG: retrieval augmented generation.

In the latest update, MetaSepsisKnowHub has evolved into an open data repository providing comprehensive coverage across various fields and precise medical knowledge of sepsis. This enhancement aims to serve intensivists and researchers by offering a clinically oriented sepsis knowledge-enhanced platform, an autonomous clinical heterogeneity analysis platform, and a potential therapeutic target screening tool. In addition, a specialized LLM framework has been developed based on RAG and prompt engineering (Figure 2), ensuring a robust and innovative platform for research and personalized clinical practice of sepsis.

#### Scenario 1: MetaSepsisKnowHub as a Knowledge-Enhanced Platform for Human Sepsis

On the Biomarkers page, MetaSepsisKnowHub provides 3 approaches to match and present sepsis biomarkers’ biomedical information: list matching, key words matching, and advanced matching. Users can input indicators significantly deviating from normal values obtained from patients, clinical cohorts, or basic experiments into the platform for matching. The platform will return all clinical evidence related to the entered indicator, including research features, descriptive information, efficacy evaluation, and abundance changes of biomarkers in patients living with sepsis. On the basis of the returned information of sample size, cutoff value, use effectiveness, and validation, users can assess whether the entered abnormal indicator can serve as a potential novel sepsis biomarker.

**Figure 3:**
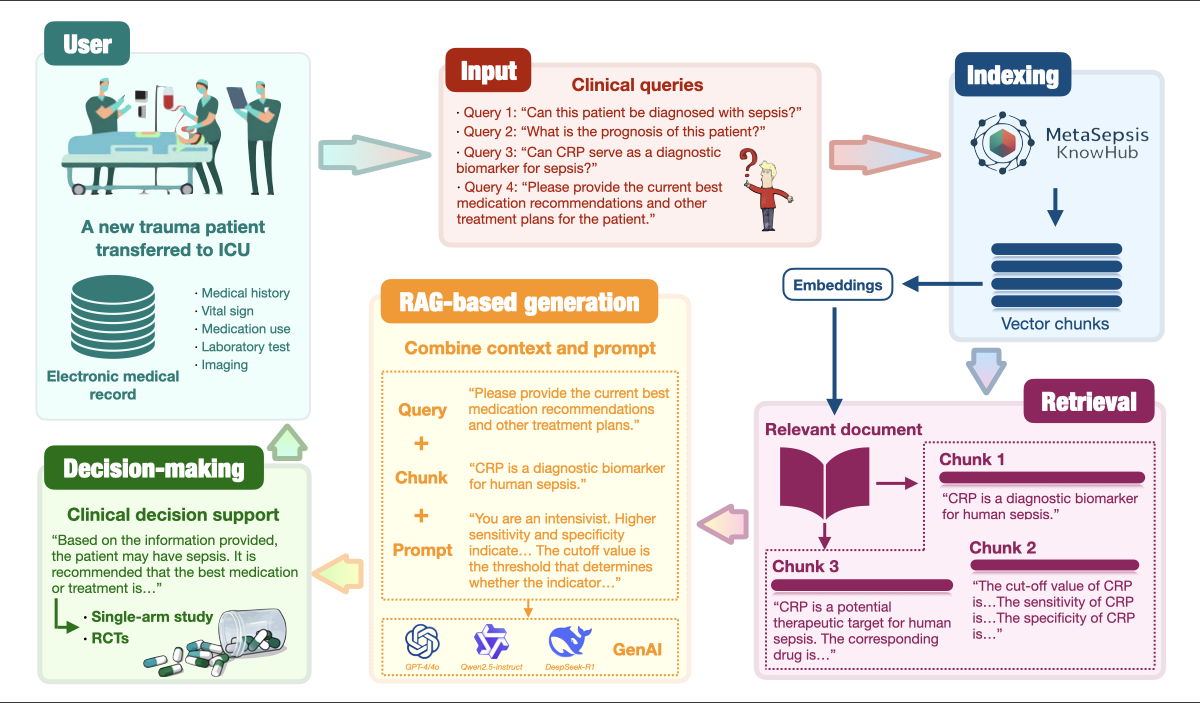
Application case for MetaSepsisKnowHub as knowledge-enhanced platform. CRP: C-reactive protein; GenAI: generative artificial intelligence; ICU: intensive care unit; RAG: retrieval augmented generation; RCT: randomized controlled trial.

In addition, we integrated MetaSepsisKnowHub with prompt engineering to construct a RAG-based LLM framework and practice the concept of knowledge enhancement. This framework enables LLMs to first retrieve relevant information from MetaSepsisKnowHub and then use the retrieved information as prompts. These prompts are compared with the patient’s actual medical history, vital signs, auxiliary examinations, medications, interventional therapies, and other clinical information to guide recommendations for sepsis diagnosis, treatment, prognosis, and other clinical decision-making processes, significantly enhancing the accuracy and stability of clinical decision support in personalized and precision medicine and the reliability of responses generated by LLMs, thereby facilitating the design and guidance of high-quality randomized controlled trials (RCTs) and single-arm studies in critical care medicine (Figure 3).

Furthermore, deep clinical phenotyping is one of the best tools for stratifying complex diseases, such as sepsis. Users can also conduct discoveries of new sepsis clinical subtypes by analyzing and combining biomedical information on sepsis biomarkers in MetaSepsisKnowHub. To enhance our understanding of the nature of sepsis, including its clinical manifestations, pathophysiology, and potential modulators, and evaluate new treatments objectively and efficiently, deep clinical phenotyping would be a prerequisite for the personalized management of sepsis.

Suppose a patient with trauma is admitted to the intensive care unit. The clinicians gather medical history, vital signs, laboratory tests, imaging, and other information to form an electronic medical record integrated into LLMs. Subsequently, clinicians can use MetaSepsisKnowHub to perform RAG, enabling LLMs to provide personalized, precise sepsis clinical decisions and, more importantly, advance high-quality RCTs and single-arm studies in critical care medicine.

#### Scenario 2: MetaSepsisKnowHub for Systems Biology and Heterogeneous Landscape of Human Sepsis

Multiple systems bioinformatics analyses can be operated in MetaSepsisKnowHub for further studies of sepsis biomarkers, representing a heterogenous landscape of human sepsis. To understand the miRNA and protein biomarkers in MetaSepsisKnowHub better in the view of systems biology, we operated network analyses in the knowledge platform. On the basis of the gene set enrichment analysis, 10,018 target genes associated with miRNA biomarkers can be identified, and there exists 4704 nodes and 6781 edges included in the miRNA-gene interaction network. The PPI network of protein biomarkers contains 147 nodes and 1753 edges.

We conducted enrichment analyses of GO Consortium and KEGG for sepsis biomarkers, including the top 20 KEGG pathways and top 5 biological processes, cellular components, and molecular functions with the lowest *P* value or false discovery rate. Interestingly, in KEGG pathway enrichment, the target genes related to miRNA sepsis biomarkers were enriched in pathways in cancer (including *ErbB* and *p53* signaling pathways) and pathways in actin cytoskeleton regulation, while protein sepsis biomarkers were mapped in infectious disease, autoimmune disease pathways, and inflammation processes. From the perspective of GO enrichment, the enrichment of target genes associated with miRNA sepsis biomarkers was observed in cellular components such as glutamate synthesis and cell surface substances [46,47]. On the other hand, the enrichment of protein sepsis biomarkers was evident in molecular functions including defense response and immune reaction.

All the results mentioned earlier and corresponding interpretations can be found on the Document page of MetaSepsisKnowHub (Table S2 and Figures S4-S6 in Multimedia Appendix 1).

#### Scenario 3: MetaSepsisKnowHub for Therapeutic Target Screening and Personalized Sepsis Management

In the era of rapid advancement in precision and personalized medicine, biomarkers have emerged as a pivotal tool, particularly in the diagnosis and treatment of infections, tumors, immune disorders, and other diseases, to guide clinicians toward the most effective treatment strategies. This approach not only aims to optimize treatment efficacy but also seeks to minimize drug toxicity and adverse effects.

MetaSepsisKnowHub systematically annotated sepsis biomarkers in the TTD and PubChem databases, aiming to identify biomarkers that could serve as potential therapeutic targets for sepsis management. The expert-curated biomedical information, along with details consisting of target names, corresponding drug names, drug categories, indications, and therapeutic classifications, is presented to users to facilitate frontier research for new drug discovery (Table S3 in Multimedia Appendix 1). Moreover, beyond aiding the screening of potential therapeutic targets, MetaSepsisKnowHub serves as a platform for the guidance of novel medications screening for sepsis, offering high-quality evidence and strategies for clinical trials.

#### Scenario 4: MetaSepsisKnowHub for Domain Knowledge Support and RAG Integration

**Figure 4:**
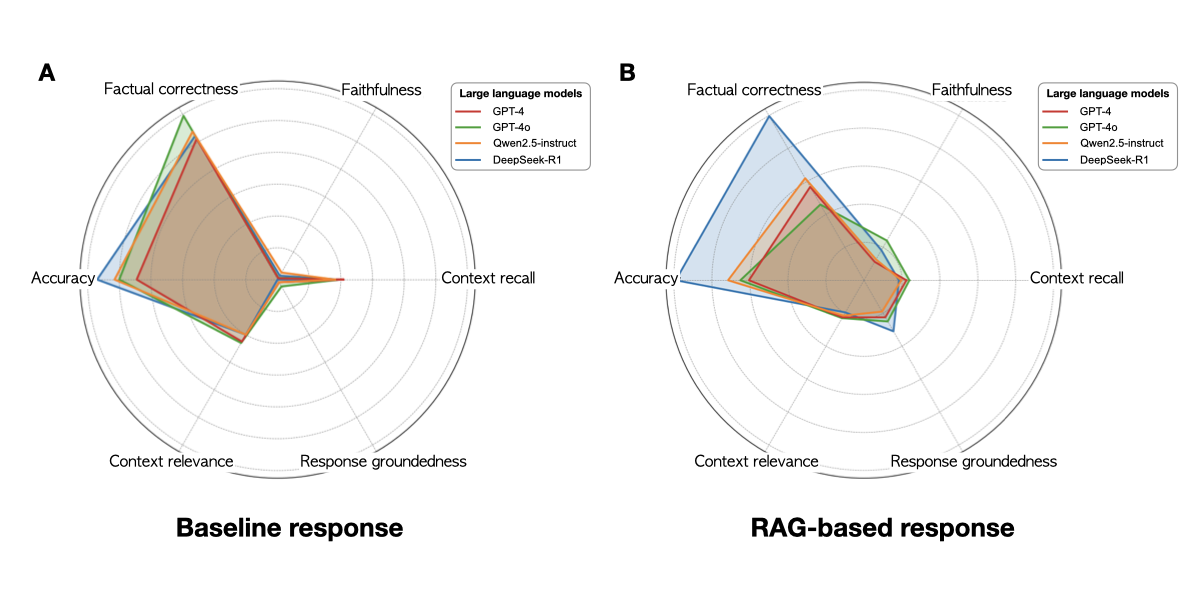
Radar charts for evaluation of retrieval augmented generation (RAG)–based responses and baselines using RAG assessments (RAGAs). (A) Radar chart for baseline responses using RAGAs and (B) radar chart for RAG-based responses using RAGAs.

We leveraged a combination of RAG based on MetaSepsisKnowHub, increasing the complexity of sepsis knowledge regarding prompt architecture and few-shot learning, and created customized RAG-based LLM frameworks applied in GPT-4, GPT-4o, Qwen2.5-instruct, and DeepSeek-R1 model environments to promote LLM tuning. We conducted a comparative validation of baseline answers generated by LLMs and answers generated by the RAG-based LLM framework, comparing them with question-answer pairs compiled based on precise knowledge and benchmark answers provided by experts using RAGAs. The RAGAs evaluation demonstrated that RAG integration significantly enhanced the factual correctness, accuracy, faithfulness, and response groundedness of LLM-generated responses, particularly benefiting DeepSeek-R1. By leveraging retrieved information, RAG improved response precision and alignment with relevant knowledge sources. However, context recall and context relevance showed a decline, indicating that while performance in accuracy improved, the LLMs may struggle to effectively preserve the contextual integrity of responses, leading to less coherent and consistent outputs in complex scenarios (Figure 4).

**Figure 5:**
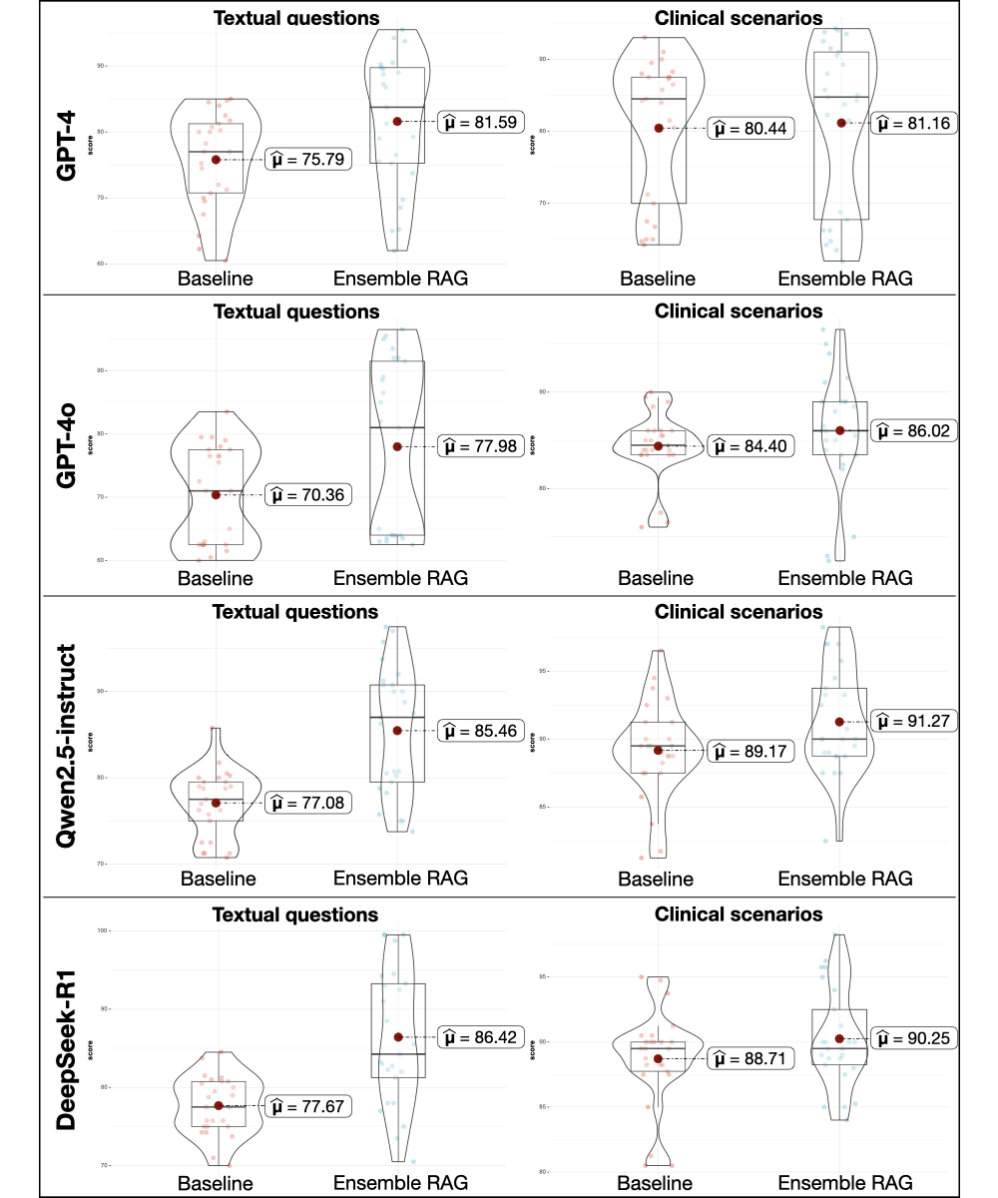
Box-plots of expert-reviewed grading evaluations for accuracy between retrieval augmented generation (RAG)–based responses and baselines.

**Figure 6:**
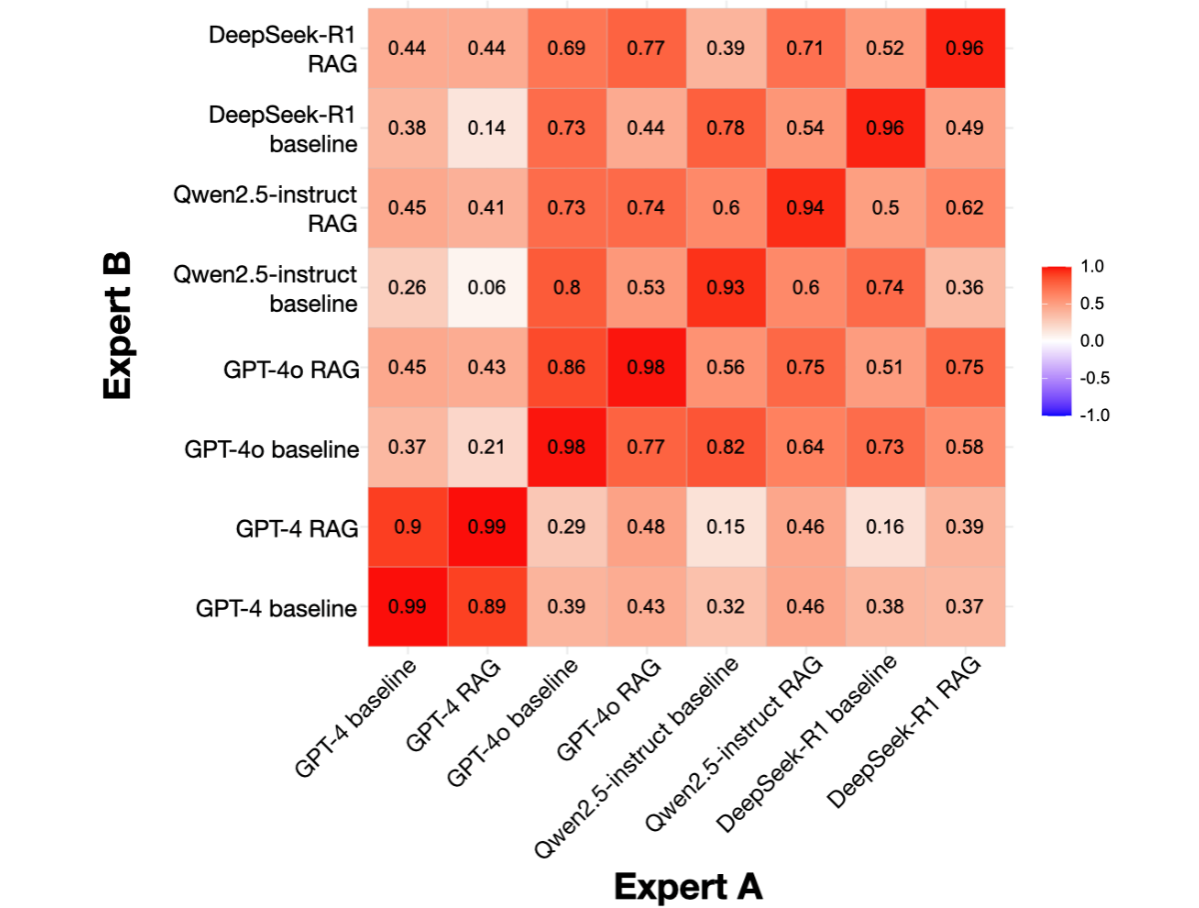
Pearson correlation coefficient heatmap for linear correlation analyses of dual expert-reviewed grading evaluations between retrieval augmented generation (RAG)–based responses and baselines. Color coding represents Pearson correlation coefficients, with red indicating positive correlation and blue indicating negative correlation. The intensity of the color reflects the strength of the correlation, ranging from –1 to +1. Pearson correlation coefficient: very strong correlation (0.8-1.0); strong correlation (0.6-0.8); moderate correlation (0.4-0.6); weak correlation (0.2-0.4); very weak correlation (0-0.2).

Expert-reviewed quantitative assessments across 4 LLMs (GPT-4, GPT-4o, Qwen2.5-instruct, and DeepSeek-R1) reveal that Ensemble RAG consistently enhances performance in both textual and clinical scenario tasks (Figure 5; Table 1). Notably, RAG-based frameworks significantly improved accuracy in textual questions over clinical scenarios, highlighting their strength in enhancing depth and precision rather than broad generalization (GPT-4: mean 75.79, SD 7.11 vs mean 81.59, SD 9.87; *P*=.02; GPT-4o: mean 70.36, SD 7.63 vs mean 77.98, SD 13.26; *P*=.02; Qwen2.5-instruct: mean 77.08, SD 3.75 vs mean 85.46, SD 7.27; *P*<.001; and DeepSeek-R1: mean 77.67, SD 3.66 vs mean 86.42, SD 8.56; *P*<.001). While clinical scenario performance also improved, lower score variance suggested a stabilizing effect in decision-making contexts. The Pearson consistency test results showed coefficients above 0.9, indicating a high level of agreement between the 2 experts’ ratings (Figure 6). The raw evaluation rating data from the 2 experts for all 400 responses is detailed in Table S4 in Multimedia Appendix 1.

The 2 experts quantitatively evaluated each LLM-generated response on a 0 to 100 scale. The group analyses comparing baseline responses with RAG-based responses demonstrated that RAG significantly enhances the depth of LLM outputs while preserving the inherent advantage of broad generalization.

Hallucinations in LLMs occur when models generate content that contradicts facts or contains logical errors. We compared baseline and RAG-based responses, analyzing hallucination frequency (Table 2). Qwen2.5-instruct and DeepSeek-R1 outperformed GPT models, exhibiting no hallucinations in either setting. GPT-4 and GPT-4o had the highest hallucination rates, particularly in textual questions, with GPT-4o performing slightly worse than GPT-4. RAG significantly reduced ICH in both textual and clinical scenarios but had no notable impact on FCH. No model exhibited CCH in clinical scenarios, suggesting that hallucinations are more prevalent in general knowledge recall than in structured reasoning. Regarding question types, clinical scenarios generate fewer hallucinations than textual questions, as they depend on extended context and intricate contextual links. LLMs leverage more domain knowledge for self-correction, minimizing unrealistic reasoning and ensuring the high accuracy and reliability essential for clinical decision-making, thus reducing hallucination risks. A total of 400 complete LLM-generated responses to 50 queries across baselines and RAG-based responses are detailed in Multimedia Appendix 1.

**Table 1:** Statistical interpretation of expert-reviewed grading evaluations of accuracy between retrieval augmented generation (RAG)–based answers and baselines.

Question type	GPT-4		GPT-4o		Qwen2.5-instruct		DeepSeek-R1	
	Textual questions	Clinical scenarios	Textual questions	Clinical scenarios	Textual questions	Clinical scenarios	Textual questions	Clinical scenarios
Quantitative gradation of baseline responses^a^, mean (SD)	75.79 (7.11)	80.44 (9.80)	70.36 (7.63)	84.40 (3.43)	77.08 (3.75)	89.17 (3.51)	77.67 (3.66)	88.71 (3.65)
Quantitative gradation of RAG-based responses^a^, mean (SD)	81.59 (9.87)	81.16 (11.42)	77.98 (13.26)	86.02 (5.92)	85.46 (7.27)	91.27 (3.75)	86.42 (8.56)	90.25 (3.73)
Change (%)	+7.65	+0.90	+10.83	+1.92	+10.87	+2.36	+11.27	+1.74
*P* value	.02	.82	.02	.25	<.001	.05	<.001	.15
Effect size^b^ (95% CI)	−0.65 (−1.21 to −0.08)	−0.07 (−0.61 to 0.48)	−0.68 (−1.24 to −0.11)	−0.32 (−0.87 to 0.23)	−1.39 (−2.01 to −0.75)	−0.56 (−1.11 to 0.002)	−1.27 (−1.89 to −0.64)	−0.4 (−0.95 to 0.15)
Qualitative inference^c^	Medium to large	Very small	Medium to large	Small to medium	Very large	Medium to large	Very large	Small to medium

^a^Two experts quantitatively evaluated each large language model–generated response on a 0 to 100 scale.

^b^The effect size was estimated with Hedges *g.*

^c^Qualitative inference: Hedges *g*=|0.2| (small effect size); Hedges *g*=|0.5| (medium effect size); and Hedges *g*=|0.8| (large effect size).

**Table 2:** Hallucination type and distribution across retrieval augmented generation (RAG)–based answers and baselines.

Large language models and question types		Fact-conflict hallucination, n (%)		Input-conflict hallucination, n (%)		Context-conflict hallucination, n (%)	
**GPT-4 baseline**							
	Textual questions (n=29)		5 (17)		24 (83)		0 (0)
	Clinical scenarios (n=2)		1 (50)		1 (50)		0 (0)
**GPT-4 RAG**							
	Textual questions (n=17)		3 (18)		14 (82)		0 (0)
	Clinical scenarios (n=1)		1 (100)		0 (0)		0 (0)
**GPT-4o baseline**							
	Textual questions (n=31)		6 (19)		23 (74)		2 (6)
	Clinical scenarios (n=2)		1 (50)		1 (50)		0 (50)
**GPT-4o baseline**							
	Textual questions (n=12)		0 (0)		12 (100)		0 (0)
	Clinical scenarios (n=3)		0 (0)		3 (100)		0 (0)
**Qwen2.5-instruct baseline**							
	Textual questions (n=0)		0 (0)		0 (0)		0 (0)
	Clinical scenarios (n=0)		0 (0)		0 (0)		0 (0)
**Qwen2.5-instruct RAG**							
	Textual questions (n=0)		0 (0)		0 (0)		0 (0)
	Clinical scenarios (n=0)		0 (0)		0 (0)		0 (0)
**DeepSeek-R1 baseline**							
	Textual questions (n=0)		0 (0)		0 (0)		0 (0)
	Clinical scenarios (n=0)		0 (0)		0 (0)		0 (0)
**DeepSeek-R1 RAG**							
	Textual questions (n=0)		0 (0)		0 (0)		0 (0)
	Clinical scenarios (n=0)		0 (0)		0 (0)		0 (0)

### Descriptive Statistical Analyses of MetaSepsisKnowHub

**Figure 7:**
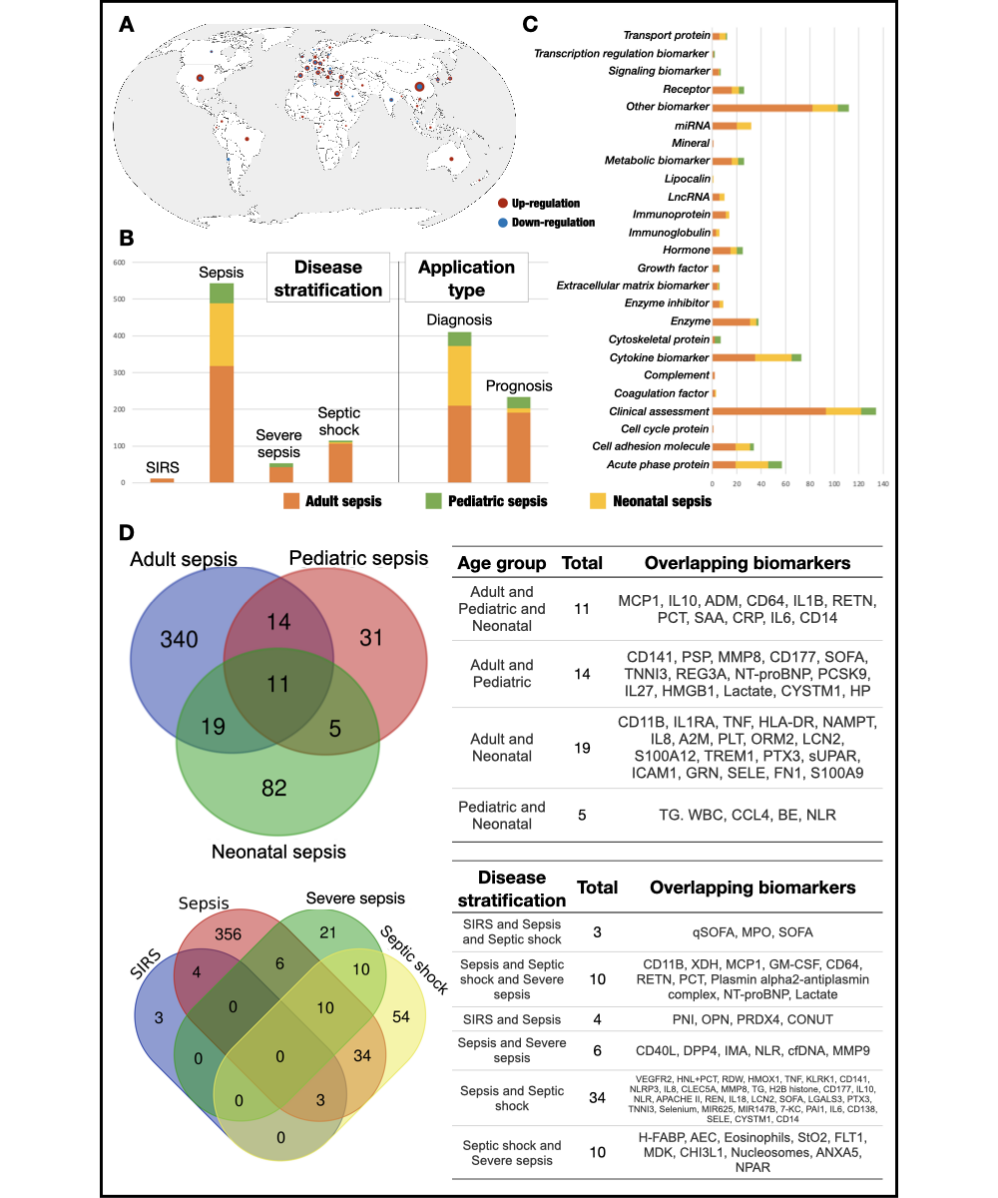
Descriptive statistical analyses results of MetaSepsisKnowHub. (A) Distribution of sepsis biomarker research worldwide in MetaSepsisKnowHub. All biomarker research in the platform were indicated on the map. The size of symbols represents the number of researches, and different colors of symbol reveals the abundance change of biomarkers; (B) bar chart of biomarker records in MetaSepsisKnowHub classified by disease classification and application type; (C) bar chart of biomarker records in MetaSepsisKnowHub classified by biomarker category; and (D) Venn diagram based on subtypes of age groups and disease stratifications, along with corresponding overlapping biomarker statistics. ADM: adrenomedullin; CD14: cluster of differentiation 14; CRP: C-reactive protein; IL6: interleukin-6; IL10: interleukin-10; Lactate: lactic acid; LncRNA: Long noncoding RNA; miRNA: microRNA; NT-proBNP: N-terminal prohormone of Brain Natriuretic Peptide; PCT: procalcitonin; qSOFA: quick Sequential Organ Failure Assessment; SIRS: systemic inflammatory response syndrome; SOFA: Sequential Organ Failure Assessment.

Relevant statistical analyses were calculated for the biomarker biomedical information, which can be found in the Document page of MetaSepsisKnowHub. We classified sepsis biomarkers in MetaSepsisKnowHub and conducted statistical analyses on different dimensions, including disease stratification, biomarker category, and application type. The knowledge platform collected sepsis biomarkers from 48 countries worldwide; the United States, Germany, and China reported most biomarkers. Moreover, the number of upregulated biomarkers in sepsis or septic shock groups was greater than that of downregulated biomarkers (Figures 7A-7C).

The Venn diagrams delineated shared and distinct biomarkers across sepsis subtypes by age group and disease severity. Overlapping biomarkers among age groups indicate common pathophysiological mechanisms, including inflammatory and immune responses (eg, interleukin-6, interleukin-1β, tumor necrosis factor, and cluster of differentiation 64) and organ dysfunction (eg, procalcitonin, Sequential Organ Failure Assessment [SOFA], and N-terminal pro B-type natriuretic peptide), reflecting age-dependent variations in immune activation. In disease severity stratification, spanning systemic inflammatory response syndrome, sepsis, severe sepsis, and septic shock, biomarker complexity and specificity increase with progression. While quick SOFA, myeloperoxidase, and SOFA scores are prevalent across early stages, advanced disease is marked by distinctive molecular signatures, including CD11B, xanthine dehydrogenase, granulocyte-macrophage colony-stimulating factor, and resistin. Furthermore, the enrichment of endothelial dysfunction (vascular endothelial growth factor receptor-2, heme oxygenase 1); immune dysregulation (CD40L, interleukin-10, and C-type lectin domain family 5 member A); and coagulation-related markers (plasminogen activator inhibitor-1 and circulating free DNA) in severe sepsis and septic shock underscores key pathways driving disease escalation (Figure 7D).

Procalcitonin, C-reactive protein (CRP), CD14, and interleukin-6 can qualify as general sepsis biomarkers by broad applicability across all sepsis subtypes, consistently reported across adult, pediatric, and neonatal cohorts, reflecting a conserved pathophysiological role. General sepsis biomarkers are pivotal for universal diagnostics, prognostics, and precision medicine, enabling standardized risk stratification and early intervention. Their recurrent presence underscores their central role in systemic inflammation, immune dysregulation, and host response, which are highly consistent with practical realities of critical care in clinical settings [48-51]. The cross-population relevance highlights core inflammatory pathways, positioning them as prime targets for biomarker-driven therapies and reinforcing their translational impact in sepsis management (Table 3).

**Table 3:** Statistical analysis of overlapping frequency of sepsis biomarkers based on age-group subtypes in different studies.

Biomarkers		Frequency of occurrence in different studies, n (%)
**Adult sepsis (N=347)**		
	PCT^a^	22 (6.3)
	SOFA^b^	14 (4)
	CD14^c^	10 (2.9)
	IL6^d^	9 (2.6)
	qSOFA^e^	8 (2.3)
	Lactate^f^	7 (2)
	MDW^g^	7 (2)
	CRP^h^	7 (2)
	S100A8^i^	6 (1.7)
	NT-proBNP^j^	5 (1.4)
	ADM^k^	5 (1.4)
**Pediatric sepsis (N=54)**		
	PCT	6 (11)
	Lactate	5 (9)
	CRP	4 (7)
	CD14	3 (6)
**Neonatal sepsis (N=126)**		
	CRP	15 (11.9)
	PCT	14 (11.1)
	IL6	10 (7.9)
	CD14	9 (7.1)
	IL10^l^	5 (4)

^a^PCT: procalcitonin.

^b^SOFA: Sequential Organ Failure Assessment.

^c^CD14: cluster of differentiation 14.

^d^IL6: interleukin-6.

^e^qSOFA: quick Sequential Organ Failure Assessment.

^f^Lactate: lactic acid.

^g^MDW: monocyte distribution width.

^h^CRP: C-reactive protein.

^i^S100A8: S100 calcium-binding protein A8.

^j^NT-proBNP: N-terminal prohormone of Brain Natriuretic Peptide.

^k^ADM: adrenomedullin.

^l^IL10: interleukin-10.

### Performance Evaluation of MetaSepsisKnowHub

**Figure 8:**
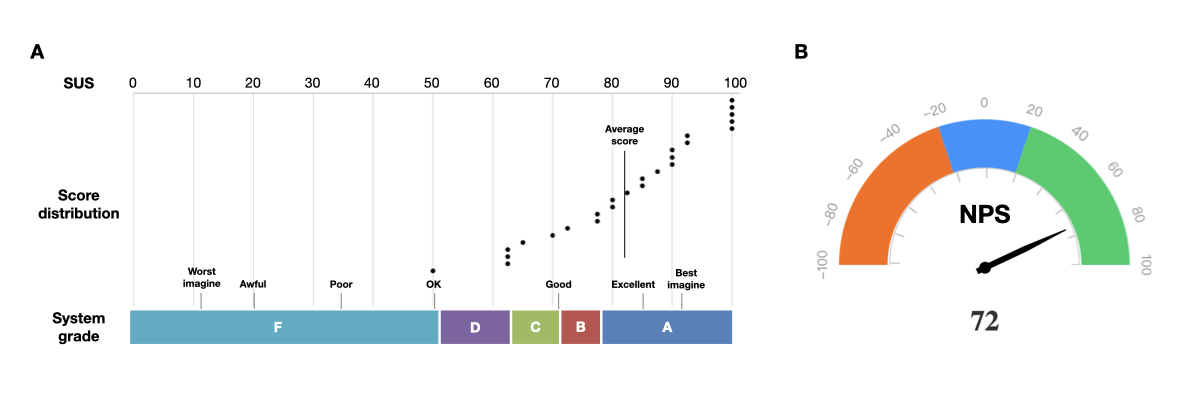
System Usability Scale (SUS) and Net Promoter Score (NPS) results for MetaSepsisKnowHub. (A) SUS score distribution and system grading of MetaSepsisKnowHub and (B) NPS of MetaSepsisKnowHub.

To verify the performances of MetaSepsisKnowHub, we conducted SUS and NPS assessments. We surveyed 25 potential users: clinicians (n=19, 76%), including intensivists (n=5, 20%); researchers (n=4, 16%); and the public (n=2, 8%). After use, the average SUS score was 82.20 (SD 14.17; Table S5 in Multimedia Appendix 1). According to system grading in the SUS assessment, MetaSepsisKnowHub was rated as “good” (Figure 8A). Of the 25 respondents, 64% (16/25) classified it as grade A, 12% (3/25) as grade B, 8% (2/25) as grade C, 12% (3/25) as grade D, and 4% (1/25) as grade F. In addition, the average accommodation score in the NPS assessments was 8.92 (SD 1.29; score range 0-10). Among respondents, 80% (20/25) were promoters (scores 9-10), 12% (3/25) were passives (scores 7-8), and 8% (2/25) were detractors (scores 0-6). The NPS score was 72, indicating high user satisfaction and loyalty (Figure 8B).

## Discussion

### Principal Findings

Biomarkers are key data in sophisticated biomedical information and are essential to the personalized diagnosis and treatment of sepsis [52]. CRP and procalcitonin have been used as clinical sepsis biomarkers for years [53,54]. However, the performance of these biomarkers has been challenged due to the low specificity of CRP and limited use of procalcitonin [55,56]. Recently, different types of biomarkers, such as tumor necrosis factor-α, IL-6, cluster of differentiation 64, and miRNAs, have shown clinical application in patients with sepsis [57-61]. Owing to the increase in the number of identified sepsis biomarkers, the time-consuming search for biomedical information extraction and personalized application has also been increasingly challenging. Furthermore, not all biomarker biomedical information has been standardized and annotated. Therefore, we updated the sepsis biomarker database to a knowledge platform, MetaSepsisKnowHub, for collection, annotation, and standardization of sepsis biomarkers. By introducing and integrating RAG and prompt engineering, we will enhance the accuracy of sepsis diagnosis and treatment recommendations generated by LLMs using precise knowledge in the knowledge platform. In addition, this platform provides researchers with a comprehensive clinical heterogeneity analysis platform, promoting the widespread application of artificial intelligence in clinical and scientific research in critical care medicine.

MetaSepsisKnowHub is as a knowledge-enhanced platform based on published sepsis knowledge. However, in the current medical domain, most knowledge-enhanced platforms still suffer from poor interpretability in reasoning processes and decision-making responses [62,63]. In addition, sepsis knowledge is vast, complex, and unstructured, with various types of data exhibiting differences in content and format, resulting in lower accuracy and usability [64]. To improve knowledge usability, it is essential to first structure and standardize sepsis knowledge while specifying the accurate semantics of knowledge in specific contexts. Therefore, while updating MetaSepsisBase, we are also developing a disease-specific ontology, Sepsis Ontology (SEPON) [65], to transform unstructured databases into an ontological knowledge platform. The construction of SEPON and standardization of sepsis knowledge could provide 3 advantages. First, users can use ontological schema to systematically organize and integrate relevant concepts, networks, or hierarchical relationships between concepts, clinical pathways, and decision processes in sepsis, thereby providing clear definitions for concepts and identifying gaps [66]. Second, ontology possesses rich semantic expressiveness and allows reasoning, enabling both humans and computers to understand it [67]. Third, ontology can trace the knowledge in MetaSepsisKnowHub, significantly increasing the interpretability of subsequent reasoning and clinical decision outcomes [68].

In the light of existing research, although some sepsis biomarkers have good diagnostic or prognostic effectiveness, none of them have been proven to work well in clinical scenarios separately [69]. Researchers and clinicians have attempted to combine different biomarkers to improve the diagnosis or prognosis of sepsis, and most of these combined biomarkers showed good accuracy (area under the curve >0.8) [70]. Through gathering all sepsis biomarkers together, MetaSepsisKnowHub provides users with an integrated platform to excavate new sepsis biomarkers and explore the combination of biomarkers for precise and personalized medicine in human sepsis. In this update, we bridged the gap between sepsis biomarkers and potential therapeutic targets, annotating biomarkers within the TTD and PubChem database to acquire and present biomedical information related to potential therapeutic drugs, nucleic acid targets, proven indications, and relevant signaling pathways. This initiative significantly broadens the clinical applicability and research scope of MetaSepsisKnowHub, contributing to establishing a sepsis-specific TTD for new drug screening and clinical trials, thereby facilitating rapid and personalized drug intervention in human sepsis [71,72].

MetaSepsisKnowHub can also support users to further investigate sepsis biomarkers at the systems biological network level. As all the biomedical information of included biomarkers was annotated by universally recognized knowledge bases, users can input sepsis biomarkers to study directly into relevant public databases including the KEGG database for signal pathway enrichment, the GO database for gene function enrichment, the STRING database for PPI network analysis, and the miRWalk database for miRNA-gene interaction network analysis.

### Comparison With Previous Work

GenAI represents the pinnacle of conversational generative LLMs, encompassing broad knowledge across various clinical domains [73]. However, the applications of GenAI in clinical settings, particularly for complex conditions such as sepsis, present notable challenges. First, there are over 8000 highly complex human diseases, and GenAI while possesses sufficient breadth of knowledge, it lacks necessary depth and precision, rendering it unsuitable for aiding clinical decision-making in complex syndromes such as sepsis [74]. Second, the rapidly evolving landscape of clinical knowledge presents a further obstacle, as traditional preprocessing models struggle to integrate continuously updated biomedical information, making GenAI models impractical for real-time clinical use [75]. Third, the lack of accuracy, interpretability, and trustworthiness in GenAI-generated recommendations remains a critical concern in critical care medicine [76]. When considering novel treatments for sepsis, clinicians require precise, evidence-based guidance, a standard that current LLMs have yet to fully meet. By integrating multiple LLMs, such as GPT-4, GPT-4o, Qwen2.5-instruct, and DeepSeek-R1, into a knowledge-enhanced platform, we can leverage RAG to ensure that diagnostic and treatment recommendations are aligned with the latest medical research and best practices, addressing the challenges outlined earlier [17].

In this study, we invited intensivists to propose potential hot topics and precise knowledge in sepsis to demonstrate the performance of a RAG-based LLM framework. We identified the framework for an LLM-friendly knowledge platform with the best application value, achieving extremely high accuracy and outperforming stand-alone LLMs in answering questions related to the clinical management of sepsis. RAG effectively enhanced the depth of responses in specialized medical domains such as sepsis, improving average precision by nearly 10% in textual questions without diminishing the broad generalization strengths of LLMs (GPT-4: +7.65%, *P*=.02; GPT-4o: +10.83%, *P*=.02; Qwen2.5-instruct: +10.87%, *P*<.001; and DeepSeek-R1: +11.27%, *P*<.001), which allowed LLMs to incorporate domain-specific knowledge, significantly boosting accuracy. In clinical scenarios, while RAG did not drastically alter accuracy, it contributed to further improvement, particularly where baseline performance was already high (GPT-4: +0.90%, *P*=.82; GPT-4o: +1.92%, *P*=.25; Qwen2.5-instruct: +2.36%, *P*=.05; and DeepSeek-R1: +1.74%, *P*=.15), suggesting that GenAI models exhibit robustness in answering complex clinical questions, achieving high accuracy across various model types in specialized medical applications. To further improve response accuracy, we must repeatedly pretrain and fine-tune prompts based on the knowledge within the platform and user queries, guiding the RAG-based LLMs to produce the desired responses. Furthermore, we evaluated the RAG-based LLM framework using the RAGAs evaluation tool and found that RAG integration significantly boosted the factual accuracy, faithfulness, and response grounding of LLMs. However, context recall and relevance declined, suggesting that while accuracy improved, LLMs struggled to maintain contextual integrity, resulting in less coherent responses in complex scenarios. The observed decline in context recall and relevance can be attributed to a few key factors. First, the RAG-based LLM framework retrieves external information to enhance factual accuracy, but this can overwhelm the LLMs with excessive or fragmented data, disrupting context recall and relevance. In complex clinical scenarios, unfiltered information may dilute the coherence of the response. Second, the retrieved data may not always align perfectly with the ongoing conversation, affecting contextual consistency. While RAG improves precision by grounding responses in external knowledge, it can sacrifice contextual integration and coherence. Third, the quality and relevance of the knowledge sources also play a crucial role, and poor retrieval optimization can further reduce contextual alignment. This underscores the necessity of comprehensive and in-depth knowledge platforms in the practice of medical AI [77,78]. In the near future, we will synchronously integrate international guidelines for management of sepsis and septic shock into the knowledge platform, and we believe that medical knowledge of sepsis will become more precise, further assisting intensivists in clinical practice and scientific research of sepsis.

The key observations regarding hallucinations are as follows. First, RAG significantly reduced hallucinations by enhancing response accuracy through external knowledge, mitigating factually unsupported content [79,80]. Second, hallucinations were more frequent with textual questions than with clinical scenarios in most LLMs. Clinical scenarios, requiring specialized medical knowledge and context, are more constrained and multidimensional, guiding LLMs to provide more accurate and grounded responses. The rigorous reasoning framework in clinical decision-making, crucial for disease diagnosis and treatment, further lowers the risk of generating false or erroneous information. Knowledge platforms like MetaSepsisKnowHub, with high credibility, help verify and self-correct generated content, further minimizing hallucinations [81]. Regarding types of LLMs, GPT-4 and GPT-4o exhibited higher hallucination rates, especially with textual questions, while Qwen2.5 and DeepSeek-R1 showed no hallucinations. ChatGPT currently stands as the most successful generative LLM based on transformer architecture [82]. Due to high model complexity and strong dependence on background knowledge, GPT-4 and GPT-4o exhibited a higher incidence of hallucinations in specialized tasks [83]. In contrast, models like Qwen2.5-instruct and DeepSeek-R1, with fewer parameters and lower complexity, exhibited minimal hallucinations on domain-specific tasks due to their limited generalization ability and weak “longtail effect” [84-86]. Finally, the distribution of hallucination types showed that FCH dominated in textual questions, which occurs when the LLMs failed to generate factually consistent content. ICH and CCH, being more specific and context dependent, are less frequent as they are better handled by the LLMs’ training data [80].

There exist a few published biomarker databases, such as the infectious disease biomarker database for infectious diseases [87], Gastric Cancer (Biomarkers) Knowledgebase for gastric cancer [88], LiverCancerMarkerRIF for liver cancer [89], and the Convention on Biological Diversity and Epigenetic Biomarker Database for colorectal cancer [90]. Compared with other biomarker databases, the advantage of MetaSepsisKnowHub is that all the data were collected from published papers by human text mining, using strict inclusion and exclusion standards. Consequently, users can conduct clinical personalized applications and scientific research related to sepsis based on this public platform. On the other hand, the design of our website is close to user needs, reducing time for users to find their required data. Moreover, MetaSepsisKnowHub is a knowledge-enhanced platform rather than a mere database, complying with SEPON and a knowledge-guided data-driven medical science paradigm to extract more precise and accurate knowledge.

Regulatory decision-making requires a thorough evaluation of all available evidence. When gaps exist, noninterventional studies can provide the real-world data (RWD) needed to support decisions. As health data become more accessible and epidemiological methods improve, real-world evidence is increasingly valuable [91]. However, while the rapid development of intelligent medicine holds promise, GenAI clinical recommendations in sepsis management cannot yet replace the rigorous, evidence-based research that informs clinical decisions. High-quality systematic reviews and meta-analyses are essential to validate GenAI outputs and ensure their accuracy. Only after such evidence is integrated can AI-generated recommendations shift from being merely supportive to genuinely beneficial for patients with sepsis, particularly in decisions about antibiotic duration, anti-inflammation, and organ dysfunctions [92-95].

Similarly, the novel sepsis biomarkers identified through MetaSepsisKnowHub require rigorous evidence-based evaluation before being considered reliable diagnostic or prognostic tools. Our future update will focus on integrating MetaSepsisKnowHub with RWD and prospective RCTs or single-arm studies to reduce the application barriers for new sepsis biomarkers. This approach will not only accelerate clinical adoption but also enhance the credibility of AI-driven recommendations, ultimately advancing sepsis care. Although the development and application of medical AI and machine learning (ML) are prioritized by academia, industry, and regulatory agencies, there is still no internationally standardized consensus on the optimal clinical study design for evaluating AI- or ML-based medical models. Key challenges include generating clinically actionable insights, mitigating algorithmic and dataset biases, and facilitating real-world integration across health care workflows and medical education [96]. The American Heart Association has emphasized that simplistic retrospective studies are often inadequate for evaluating the prognostic value of AI or ML in cardiovascular care. It calls for interdisciplinary collaboration between clinicians and data scientists, the adoption of causal inference frameworks, and careful consideration of institutional and temporal heterogeneity in electronic medical records to improve model validity and generalizability [97]. A recent RCT [98] involving more than 20,000 oncology patients found that AI-driven notifications to oncologists, identifying disease progression and suggesting genomically matched clinical trials, did not significantly improve trial enrollment, despite high model accuracy (area under the receiver operating characteristic=0.85). This highlights that accurate disease progression prediction and automated trial matching alone are insufficient to enhance participation rates. The real-world effectiveness of AI interventions requires rigorous clinical validation, and addressing information asymmetry alone may not overcome enrollment barriers. Moreover, the impact of AI tools is highly context dependent, and misidentifying the core problem or misaligning intervention goals can limit outcomes. Importantly, trial recruitment is often hindered by systemic and behavioral factors (such as referral patterns, geographic or financial constraints, and patient attitudes) rather than matching inefficiencies alone. To truly optimize clinical trial enrollment, AI must extend beyond technical matching and address broader challenges across patient populations and clinical workflows [98,99].

Compared to RCTs and retrospective studies, single-arm prospective studies are emerging as a viable and increasingly accepted alternative for evaluating AI- or ML-based medical models or clinical decision support systems (CDSSs). While RCTs offer methodological rigor, their rigid design, high cost, and long duration often limit their adaptability to fast-evolving AI technologies that require iterative refinement. Retrospective studies, on the other hand, are constrained by data heterogeneity and inherent biases, which can undermine the evaluation of real-time clinical performance. In contrast, single-arm studies provide a more flexible yet prospective framework that enables timely assessment of AI interventions in real-world settings. Regulatory bodies such as the Food and Drug Administration and European Medicines Agency have recognized the value of single-arm trials, especially when paired with RWD to construct external comparative arms [100,101]. Notably, recent trends in oncology, such as in multiple myeloma research, reflect a growing shift toward this design [102]. For AI-driven platforms, which rely on dynamic data and often lack the fixed intervention structure of traditional therapies, single-arm studies augmented by high-quality RWD may offer a more appropriate and impactful evaluation strategy than conventional RCTs.

### Limitations and Improvements

In the process of establishing and updating the platform, we carried out a series of standardized and strict screening on the data and paid more attention to the biomarkers with significant statistical significance and clinical value in sepsis.

First, we selected the PubMed and Web of Science databases as the primary sources for sepsis biomarker data due to their comprehensive coverage in the medical field. To avoid duplication, we excluded other databases such as ScienceNet, Scopus, EBSCO, OVID, and Embase, which may have led to some gaps and omissions in the data. In the future, we will expand the literature retrieval scope to include additional sources for a more complete dataset.

Second, the current limitations of MetaSepsisKnowHub include the potential for human error, despite regular checks and the time-consuming process of manually screening all data. As MetaSepsisKnowHub is still in its early stages with a relatively small dataset, manual data collection has proven more efficient than natural language processing (NLP) methods, which require further optimization and high-quality data annotation to ensure reliable results. Furthermore, manual data collection, particularly when verified by multiple reviewers, provides greater accuracy and certainty than current NLP techniques, which are still being refined. To address these challenges, future updates will introduce semiautomated data extraction methods, combining manual collection with advanced AI techniques including NLP, ontology, classification systems, topic mapping, and logical models. This integration will enable more efficient conversion of unstructured knowledge into semistructured or structured formats, striking a balance between efficiency and accuracy.

Third, the current positioning of MetaSepsisKnowHub is a knowledge-enhanced platform of sepsis biomarkers for personalized sepsis management. In future updates, MetaSepsisKnowHub will undergo continuous, multilayered integration, including the incorporation of RWD to uncover potential objective disease patterns beyond the existing literature. Regarding clinical application, we will integrate practical web-based research tools for bioinformatics and systems analyses, including RAG frameworks, enrichment analysis, and association analysis, transforming the platform into a truly comprehensive CDSS for sepsis management, and implement caching mechanisms (eg, Redis) to optimize frequent queries or retrieval requests [103].

**Figure 9:**
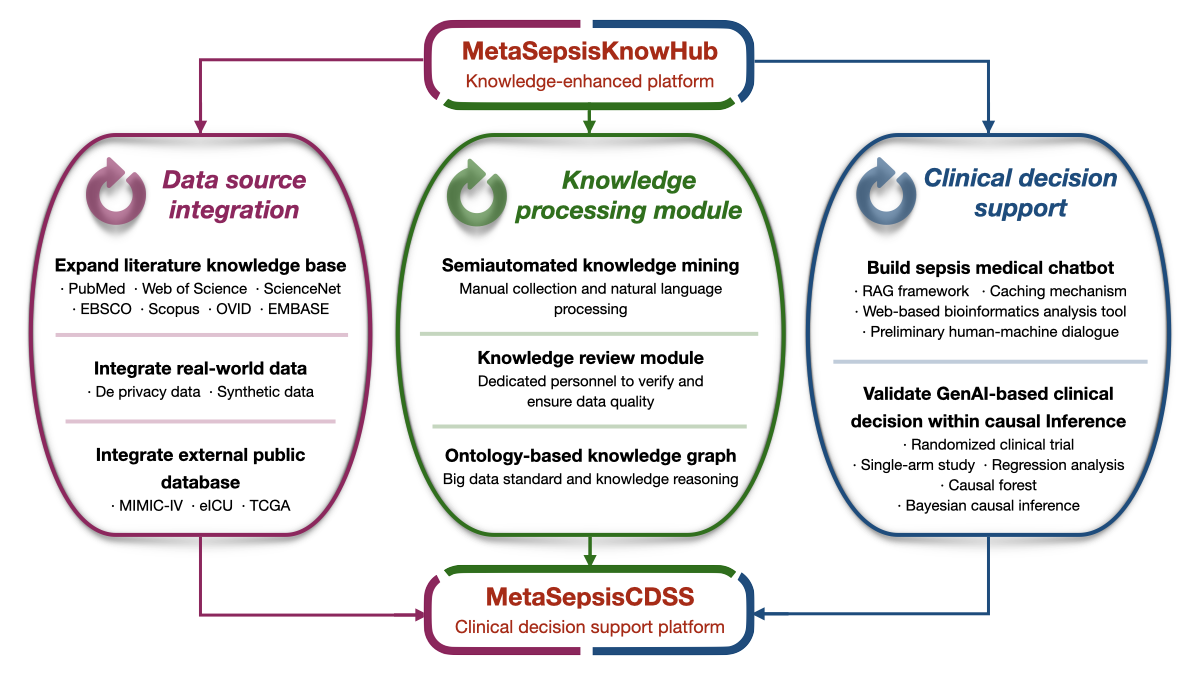
The future landscape of continuous updates and improvements of MetaSepsisKnowHub. CDSS: clinical decision support system; eICU: electronic intensive care unit Collaborative Research Database; GenAI: generative artificial intelligence; MMIC-IV: Medical Information Mart for Intensive Care-IV; RAG: retrieval augmented generation; TCGA: The Cancer Genome Atlas Program.

Finally, and most importantly, we will leverage graph data science to extract “entity-relationship-entity” triples from the extensive knowledge within the platform, constructing a comprehensive knowledge graph for sepsis. This will enable the reasoning and integration of complex knowledge systems, facilitating natural language–based decision-making for sepsis diagnosis and treatment, as well as supporting new drug discovery recommendations. In parallel, we will explore GraphRAG research to further enhance the platform’s capabilities [104]. We will also design a sepsis medical chatbot within MetaSepsisKnowHub, promoting user-friendliness for patients and the public and forwarding seamless communication capabilities [105]. The chatbot will expand the diverse clinical applications of the knowledge platform in sepsis diagnosis, treatment, prognosis, prevention, and patient education, revolutionizing the clinical practice of critical care medicine. However, it is important to note that the safety of medical AI has not yet been widely accepted in the academic community [106]. Therefore, any GenAI-driven medical interventions must be conducted under rigorous causal inference models, such as RCTs, single-arm studies, propensity score matching, and structural equation modeling, and within a strict ethical and legal framework [107]. Clinical practitioners must exercise caution when relying on GenAI-generated outputs, ensuring thorough validation before applying them in practice (Figure 9).

Future updates and improvements of MetaSepsisKnowHub will prioritize the integration of diverse data sources, the development of more automated and intelligent data-processing modules, and the implementation of a causality-driven framework for interpretable sepsis clinical decision-making. These advancements aim to evolve the knowledge-enhanced platform into a fully functional CDSS.

### Conclusions

MetaSepsisKnowHub is the first knowledge-enhanced platform for sepsis. Our goal is to extract reported sepsis biomarkers to provide users with comprehensive biomedical information and integrate RAG and prompt engineering to enhance the accuracy, stability, and interpretability of clinical decisions recommended by LLMs. MetaSepsisKnowHub not only provides an integrated platform with annotated knowledge that can be used directly to determine functions of single or combined biomarkers, but also supplies information for biological systems analysis to conduct deep phenotyping and understand clinical heterogeneity in human sepsis, as well as publicly reported biomedical knowledge resources that enable researchers and clinicians to discover complicated translational associations for new drug discovery for sepsis. In the near future, based on ontological data schema and MetaSepsisKnowHub, we will structure a knowledge graph and medical chatbot for sepsis to enable the extraction, reasoning, and integration of complicated knowledge, further facilitating more accessible, intelligent, and personalized applications for full course management of human sepsis.

## Data Availability

All queries and retrieval augmented generation–based large language model answers are included in Multimedia Appendix 1, and all biomedical information of sepsis biomarkers can be found on the Download page of the MetaSepsisKnowHub website [21]. For any additional information, please contact the corresponding authors.

## Appendix

Multimedia Appendix 1 Supplementary materials for MetaSepsisKnowHub, including literature retrieval methods, platform architecture, bioinformatics analyses, retrieval augmented generation–based question-answer system design, and evaluation results.

## Abbreviations

API: application programming interface
CCH: context-conflict hallucination
CDSS: clinical decision support system
CRP: C-reactive protein
FCH: fact-conflict hallucination
GenAI: generative artificial intelligence
GO: Gene Ontology
ICH: input-conflict hallucination
KEGG: Kyoto Encyclopedia of Genes and Genomes
LLM: large language model
miRNA: microRNA
ML: machine learning
NLP: natural language processing
NPS: Net Promoter Score
PPI: protein-protein interaction
RAG: retrieval augmented generation
RAGAs: retrieval augmented generation assessments
RCT: randomized controlled trial
RWD: real-world data
SEPON: Sepsis Ontology
SOFA: Sequential Organ Failure Assessment
SUS: System Usability Scale
TTD: Therapeutic Target Database
